# Anticoagulant Activity of Sulfated Ulvan Isolated from the Green Macroalga *Ulva rigida*

**DOI:** 10.3390/md17050291

**Published:** 2019-05-14

**Authors:** Amandine Adrien, Antoine Bonnet, Delphine Dufour, Stanislas Baudouin, Thierry Maugard, Nicolas Bridiau

**Affiliations:** 1 Equipe BCBS (Biotechnologies et Chimie des Bioressources pour la Santé), La Rochelle Université, UMR CNRS 7266 LIENSs, Avenue Michel Crépeau, 17042 La Rochelle, France; amandine.adrien@seprosys.com (A.A.); antoine.bonnet@univ-lr.fr (A.B.); thierry.maugard@univ-lr.fr (T.M.); 2 SEPROSYS, Séparations, Procédés, Systèmes, 12 Rue Marie-Aline Dusseau, 17000 La Rochelle, France; delphine.dufour@seprosys.com (D.D.); stanislas.baudouin@seprosys.com (S.B.)

**Keywords:** *Ulva rigida*, ulvan, chemical sulfation, anticoagulant activity

## Abstract

(1) Background: Brown and red algal sulfated polysaccharides have been widely described as anticoagulant agents. However, data on green algae, especially on the *Ulva* genus, are limited. This study aimed at isolating ulvan from the green macroalga *Ulva rigida* using an acid- and solvent-free procedure, and investigating the effect of sulfate content on the anticoagulant activity of this polysaccharide. (2) Methods: The obtained ulvan fraction was chemically sulfated, leading to a doubling of the polysaccharide sulfate content in a second ulvan fraction. The potential anticoagulant activity of both ulvan fractions was then assessed using different assays, targeting the intrinsic and/or common (activated partial thromboplastin time), extrinsic (prothrombin time), and common (thrombin time) pathways, and the specific antithrombin-dependent pathway (anti-Xa and anti-IIa), of the coagulation cascade. Furthermore, their anticoagulant properties were compared to those of commercial anticoagulants: heparin and Lovenox^®^. (3) Results: The anticoagulant activity of the chemically-sulfated ulvan fraction was stronger than that of Lovenox^®^ against both the intrinsic and extrinsic coagulation pathways. (4) Conclusion: The chemically-sulfated ulvan fraction could be a very interesting alternative to heparins, with different targets and a high anticoagulant activity.

## 1. Introduction

Marine macroalgae are used in several industrial applications and have represented a sharply increasing annual market over the last decades, going from about USD 6 billion in 2003 to currently USD 10.6 billion, and exhibiting an annual growth rate close to 10%, due to the high expansion of the aquaculture sector, which represents today 97% of the global seaweed production worldwide [1,2,3]. Seaweed and seaweed products are mainly used for human consumption (85%) and in the hydrocolloid industry. The three main groups of marine algae (Phaeophycae, Rhodophyta, and Chlorophyta) are rich sources of various compounds of interest such as proteins, pigments, and polysaccharides, whose potential market is estimated at several USD billion [3,4,5], and have been studied for their potential pharmaceutical, cosmetic, or nutraceutical applications. Polysaccharides from brown and red marine macroalgae are widely used in the industry for their gelling properties, alginates, agar, and carrageenans in particular [1,3]. It has also been shown that sulfated polysaccharides from marine macroalgae have numerous biological activities, including immunoinflammatory [6,7,8], antioxidant [9,10], antitumor [11,12,13], antiviral [7,14,15,16], and anticoagulant [17,18,19,20] properties. Conversely, although they have been consumed for centuries, green macroalgae are still relatively unexploited. Nevertheless, the discovery of ulvans, the sulfated polysaccharides from green algae of the *Ulva* genus (including species from the formerly genus *Enteromorpha*), has increased the interest for these seaweeds. Ulvans are mainly composed of rhamnose and uronic acids (glucuronic or iduronic acid) [21,22]. The main repeated disaccharide units of ulvans are [→4)-*β*-d-Glc*p*-(1→4)-*α*-l-Rha*p*3S-(1→]_n_ (type A) and [→4)-*α*-l-Ido*p*-(1→4)-*α*-l-Rha*p*3S-(1→]_n_ (type B) [7].

Heparin, a polysaccharide belonging to the glycosaminoglycan family, is mainly composed of l-iduronic-2-*O*-sulfate acid and d-glucosamine-*N*-sulfate, 6-*O*-sulfate. Unfractionated heparin (UFH) has an average molecular weight (MW) of 15 kDa [23]. Its anticoagulant activity is primarily due to its specific antithrombin-binding pentasaccharide sequence, where the central glucosamine is not only 2- and 6-*O*-sulfated but also 3-*O*-sulfated [24]. Despite of its major anticoagulant activity, UFH may cause serious adverse events (AE), such as heparin-induced thrombocytopenia [25] or hemorrhage [26]. Moreover, its low bioavailability [27] makes such a treatment really expensive. To reduce the risks of AE, heparin may be depolymerized to produce smaller molecules, known as low-molecular-weight heparins (LMWH, MW_avg_ ≥ ∼6 kDa) [28]. Although LMWH significantly reduce the risks of AE associated with UFH, their recommended use as first-line treatment for cancer-associated venous thromboembolism (VTE), for at least 3 to 6 months, still exposes patients to allergic reactions, heparin-induced thrombocytopenia, recurrent VTE, and major bleeding events, leading to high rates of treatment discontinuation [29,30]. Given the risks and high costs of these treatments, there is a compelling need for further investigations to identify new sources of anticoagulants.

The first studies assessing the anticoagulant activity of sulfated polysaccharides from macroalgae have shown that polysaccharides from brown algae could be alternative sources of new anticoagulant compounds. Sulfated fucoidans from brown algae and sulfated galactans from red seaweeds (also referred to as carrageenans) seem to have a strong anticoagulant activity. Data on the anticoagulant activity of polysaccharides from green macroalgae are limited compared to brown and red algae, but a few studies have demonstrated their potential. Regarding the anticoagulant activity, the most studied genera are *Codium* [31,32,33,34,35,36], which contains sulfated arabinans and arabinogalactans, and then *Monostroma*, which contains sulfated rhamnans [37,38,39,40,41,42]. Only two studies have shown the potential anticoagulant properties of ulvans extracted from *Ulva conglobata* and *Ulva reticulata* [43,44].

The relationship between the polysaccharide chemical structure and the anticoagulant activity is complex but previous studies have shown that several factors, such as MW, osidic composition, and sulfate content and substitution pattern, may significantly affect the anticoagulant activity [17,45,46,47]. Polysaccharide sulfate content appears to have a major impact on its anticoagulant potential. Thus, Cianca et al. have demonstrated that sulfated galactans extracted from *Codium fragile* and *Codium vermilara* with a high MW and high sulfate content have a higher anticoagulant activity than LMW and low sulfate content polysaccharides [36]. Furthermore, the only published studies that have highlighted a strong activity of sulfated rhamnans from the *Ulva* genus were based on highly sulfated ulvans (sulfate content of 26–35%) [43,44].

In this study, a solvent- and acid-free process to extract and purify ulvan from *Ulva rigida* was developed. To study the effect of sulfate content, a chemical sulfation procedure based on the sulfur-trioxide pyridine complex method was performed on the ulvan fraction obtained. The anticoagulant activity of both the native and chemically-sulfated ulvan fractions was then assessed against the intrinsic and/or common (activated partial thromboplastin time: APTT), extrinsic (prothrombin time: PT), and common (thrombin time: TT) pathways, and the specific antithrombin-dependent pathway (anti-Xa and anti-IIa), of the coagulation cascade.

## 2. Results and Discussion

### 2.1. Extraction, Purification, and Sulfation of Ulvan from U. rigida

The extraction of ulvan from the green macroalga U. rigida was carried out in hot water and was followed by a multistep purification procedure previously developed [48]: ultrafiltration, ion exchange, and precipitation of remaining proteins. After neutralization with 1 M NaOH, the sulfated polysaccharides were recovered in a sodium salt form in the ULVAN-01 fraction, with an extraction yield of about 5% of the non-desalinated dry biomass (initially containing about 50% of salt).

The sulfation procedure was finally carried out starting from 500 mg of ULVAN-01 fraction, to give 200 mg of ULVAN-02 fraction.

### 2.2. Chemical Characterization of the ULVAN-01 and ULVAN-02 Fractions

The efficacy of the extraction and purification procedure of ulvan from *U. rigida* in terms of sulfated polysaccharide production was high with less than 4% of remaining contaminant proteins in the ULVAN-01 fraction. The purity of the polysaccharide was even higher after chemical sulfation with a percentage of remaining proteins less than 1% in the ULVAN-02 fraction (Table 1). This better purification was very likely due to the last step of the sulfation procedure, i.e., ethanol precipitation of sulfated polysaccharides.

Both ulvan fractions contained about 30% of uronic acids, a value that was in the higher range of what could be observed in ulvans from *Ulva* species [49]. Conversely, the sulfate content of about 11%, corresponding to a sulfate to uronic acid molar ratio of 0.65, was in the lower range of ulvan sulfate content [22,49]: this could be explained by the harsh conditions of extraction, ultrafiltration and protein precipitation used during the isolation process (80 °C, 5 bars), which may have led to partial desulfation. ULVAN-01 was mainly composed of rhamnose and glucuronic and/or iduronic acid, and to a lesser extent of glucose and/or galactose, xylose, and glucosamine. It is noteworthy, however, that chemical sulfation allowed to almost double the initial sulfate content of ULVAN-01: indeed ULVAN-02 exhibited 20% of sulfate groups on the polysaccharide backbone, for a sulfate to uronic acid molar ratio of 1.30. Moreover, a very interesting feature of the extraction procedure developed by SEPROSYS was the high purity of the resulting ulvan in the ULVAN-01 fraction, which was estimated at 86% by adding the contents of neutral sugars, uronic acids, and sulfate esters, taking into account that sulfates are not burned at 550 °C and are also part of the ash content, together with sodium cations.

### 2.3. Structural Characterization of the ULVAN-01 and ULVAN-02 Fractions

**Figure 1:**
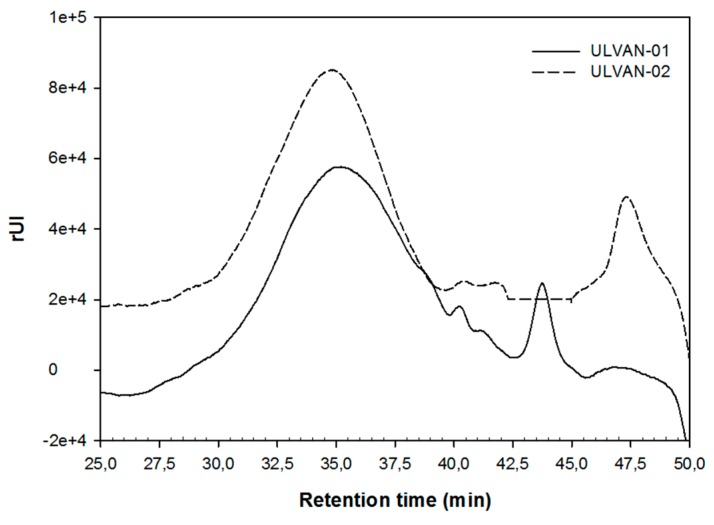
Size exclusion chromatograms of the ULVAN-01 and ULVAN-02 fractions.

A M_W_ of about 57 kDa was estimated by SEC (Figure 1 and Table 1) for ULVAN-01, based on a calibration curve of dextran standards. After chemical sulfation of ULVAN-01 using sulfur trioxide pyridine complex in pyridine and dimethylformamide (DMF), the resulting ulvan in ULVAN-02 had an estimated M_W_ of about 55 kDa, slightly lower than ULVAN-01 M_W_. SEC chromatograms of ULVAN-01 and ULVAN-02 were very similar, except that the major peak eluted from 30 to 40 min seemed narrower for ULVAN-02 and eluted slightly sooner than that of ULVAN-01. On the other hand, a peak eluted at about 44 min, corresponding to oligosaccharides, was only found in ULVAN-01, while a peak eluted at about 47 min, likely corresponding to low-molecular-weight oligosaccharides, was only found in ULVAN-02. Besides, the polydispersity of ULVAN-02 (I = 1.4) was lower than that of ULVAN-01 (I = 1.8). The fact that the sulfate content in ULVAN-02 was double to the respective measured in ULVAN-01 should highly affect M_W_, considering the hydrodynamic size of the polysaccharide that is strongly affected by sulfate groups, due to anionic charges. Therefore, the weak difference between ULVAN-01 and ULVAN-02 M_w_ may appear very surprising. However, these observations could be explained by a slight cleavage of the glycosidic linkages in the polysaccharides that likely occurred during chemical sulfation, as proposed by Nishino and Nagumo [50]. This would lead to the transformation of bigger polysaccharides into polysaccharides of intermediary MW, and therefore to the increase in M_n_ observed in ULVAN-02, concomitant with the decrease in polydispersity index, resulting in the narrower shape of the major peak eluted from 30 to 40 min. This would also cause the depolymerization of oligosaccharides to smaller oligosaccharides, which might explain the appearance of the peak eluted at 47 min for ULVAN-02 as the peak eluted at 44 min found in ULVAN-01 disappeared. Furthermore, this would be in accordance with the slightly lower value of ULVAN-02 M_w_ as small oligosaccharides strongly affect the M_w_ value.

ULVAN-01 was also characterized in a previous work by ^13^C NMR [48]. The chemical shifts were attributed on the basis of references reporting assignments of ulvans and oligosaccharides [51]. As previously reported, the assignment signals corresponding to the carbons of both type A (*β*-d-Glc*p*-(1→4)-*α*-l-Rha*p*3S) and type B (*α*-l-Ido*p*-(1→4)-*α*-l-Rha*p*3S) ulvanobiouronic acid 3-sulfate were detected, confirming the presence of this sequence. On the other hand, the ultra high pressure liquid chromatography-high resolution mass spectrometry (UHPLC-HRMS) analyses of the 9kDa ulvan fraction obtained after controlled depolymerization of ULVAN-01 revealed characteristic ions that could be attributed after selective pseudo-MS^3^ fragmentation to oligosaccharide sequences with a polymerization degree up to 12 (Table 2).

Sequences 1 to 4 were determined by pseudo-MS^3^ analysis and shown to exhibit a repeated disaccharide sequence, sulfated or not, composed of a rhamnose moiety and an uronic (glucuronic or iduronic) acid, i.e., ulvanobiouronic acid 3-sulfate [48]. However, this typical structure of ulvans was found associated to 3 variable moieties, despite being quite uncommon: glucose (or possibly a galactose since these epimers cannot be distinguished by MS/MS analysis) (sequence 3), xylose (sequence 4), or uronic acid (sequence 1). Together, ^13^C NMR and MS analyses were thus in line with the biochemical and monosaccharide compositions of ULVAN-01, which put forward a high proportion of neutral sugars (mainly rhamnose) and uronic acids. Glucosamine was not found in these products of ulvan-01 depolymerization.

**Scheme 1:**
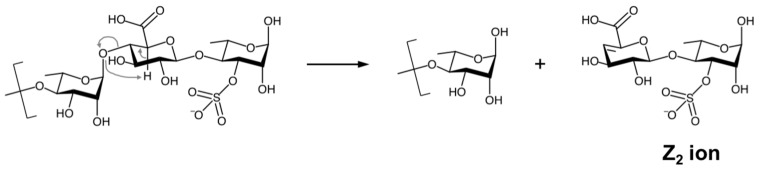
Dissociation mechanism resulting in Z_2_ ion formation (example of the ion (*m/z*) 401.0385: sequence 5 in Table 2 and Figure 2A) (adapted from [53]).

The MS^2^ analyses also revealed four disaccharide sequences in ULVAN-02 that were characterized as Z_2_ ions (*m/z*) obtained after fragmentation of the polysaccharidic chain in the spectrometer source, in line with the work by Saad and Leary [53], who observed the same dissociation mechanism when analyzing heparin disaccharides by electrospray ionization MS^2^ analysis. The dissociation mechanism of ULVAN-02 giving access to the Z_2_ ion (*m/z*) 401.0385 (sequence 5 in Table 2) is shown as an example on Scheme 1. The regio- and stereo-specificities of the osidic linkages could not be determined from these analyses so that the reported structures were designed according to the established structure of ulvans [22].

**Figure 2:**
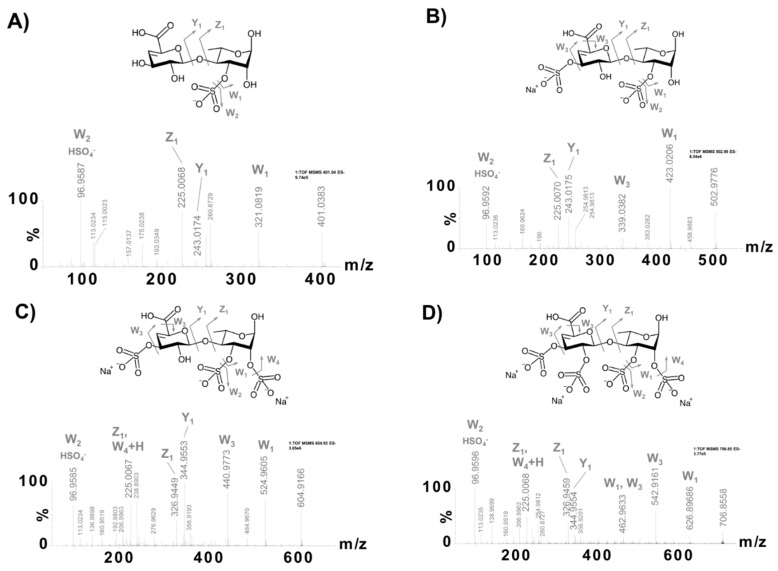
Negative-ion mode electrospray ionization-mass spectrometry targeted fragmentation spectra (ESI^−^-pseudo-MS^3^) of the ions (*m/z*) 401.0383 (**A**, sequence 5 in Table 2), 502.9776 (**B**, sequence 6 in Table 2), 604.9166 (**C**, sequence 7 in Table 2), and 706.8558 (**D**, sequence 8 in Table 2).

The fragmentation profiles of the four disaccharide sequences obtained after pseudo-MS^3^ analysis proved that these sequences exhibited sulfate groups on both rhamnose and uronic acid moieties, with one to two sulfate groups on each residue (sequences 5–8 in Table 2 and Figure 2). This result demonstrated that the chemical sulfation method used was completely random and could sulfate up to all four free alcohols of the ulvanobiouronic acid disaccharide: two of rhamnose and two of uronic acid (GlcA or IdoA). Besides, given both the sulfate content of ULVAN-02 of about 20%, indicating an average sulfation of two sulfate groups every three residues, and the observation of disaccharide sequences with up to four sulfate groups, it was concluded that the chemical sulfation of the ULVAN-01 polysaccharide led to a high heterogeneous sulfation pattern, involving both hyper-sulfated and scarcely sulfated zones within ULVAN-02 polysaccharidic structure.

### 2.4. Anticoagulant Activity

The potential anticoagulant activity of ULVAN-01 and ULVAN-02 was investigated using different clotting and enzymatic assays, targeting the intrinsic and/or common (activated partial thromboplastin time, APTT), extrinsic (prothrombin time, PT) or common (thrombin time, TT) pathways of the coagulation cascade, as well as the specific antithrombin-dependent pathway (anti-Xa and anti-IIa). Furthermore, the anticoagulant properties of ulvan fractions were compared to those of commercial anticoagulants, one UFH and one LMWH, the so-called Lovenox^®^.

**Figure 3:**
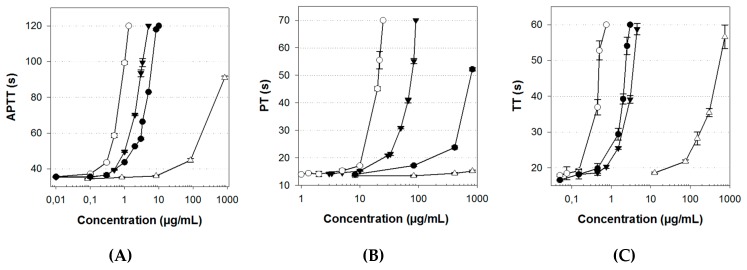
(**A**) Activated partial thromboplastin time (APTT), (**B**) prothrombin time (PT), and (**C**) thrombin time (TT) assays of ULVAN-01 (Δ), ULVAN-02 (▼), unfractionated heparin (UFH) (○), and Lovenox^®^ (●). The clotting times of the plasma in the absence of fractions (negative control, 0.9% NaCl) were (**A**) 38.2 s, (**B**) 13.1 s, and (**C**) 12.9 s. The maximum clotting times measured by the coagulometer were (**A**) 120 s, (**B**) 70 s, and (**C**) 60 s (no coagulation within this time range). Data shown as the mean +/−SD, n = 6.

The APTT test mainly assesses the anticoagulant activity on the intrinsic pathway, and, to a lesser extent, on the common pathway. ULVAN-01 activity on the intrinsic pathway was very low (Figure 3A). Indeed, at a concentration of 1000 µg/mL, the clotting time of the plasma was less than 100 s, i.e., 100- and 1000-fold higher than that of Lovenox^®^ and UFH, respectively. Conversely, ULVAN-02 showed a very interesting anticoagulant activity that was higher than that of Lovenox^®^ and just slightly lower than that of UFH. While 500 µg/mL of ULVAN-01 were needed to double the clotting time, only 2.4 µg/mL of ULVAN-02 gave the same result: this concentration was two-fold lower than that of Lovenox^®^ (4.7 µg/mL) and only three-fold higher than that of UFH (Table 3). ULVAN-02 appeared thus to be more effective than Lovenox^®^ on the intrinsic pathway.

Therefore, the chemical sulfation procedure significantly increased the activity of ULVAN-01 on the intrinsic and/or common coagulation pathways. Several sulfated polysaccharides from green macroalgae species have been studied for their anticoagulant activity, including high arabinose-containing sulfated polysaccharides, arabinogalactans, or galactans found in *Codium* [32,34,36] or *Ulva* (formerly *Enteromorpha*) [54,55]*;* polysaccharides rich in sulfated galactose from *Caulerpa* [56]; and high-rhamnose-containing sulfated polysaccharides from *Monostroma* [38,40,41,57]. It has mainly been shown that these polysaccharides could extend the APTT. Only one study has assessed, by APTT assay, a significant anticoagulant activity of sulfated ulvans extracted from an *Ulva* species (*Ulva conglobata*) [43]. These polysaccharides were naturally highly sulfated (between 23.0 and 35.2%), with a rhamnose content between 63 and 72%. The most effective ulvan contained 35.2% of sulfates and prolonged the clotting time to 120 s at a concentration of 2.5 µg/mL, versus 4 µg/mL and 5 µg/mL for two other ulvans with 23 and 28% of sulfate content, respectively. Interestingly, the concentration of 4 µg/mL in ULVAN-02 (sulfated at 20%) resulted in the same clotting time, thus making its activity very similar to that of *Ulva conglobata* ulvans.

The PT test, assessing the ulvan activity on the extrinsic coagulation pathway, also showed a major effect of the sulfation procedure on the polysaccharide activity. Indeed, ULVAN-01 was inactive on this coagulation pathway whereas the activity of the sulfated fraction ULVAN-02 was high and only three-fold lower than that of UFH (Figure 3B). ULVAN-02 activity was more than ten-fold higher than that of Lovenox^®^, with respective concentrations needed to double the clotting time of 45 and 480 µg/mL (Table 3).

Furthermore, a significantly increased anticoagulant activity was associated with ULVAN-01 sulfation, based on the TT assay, which evaluates the anticoagulant activity on the common pathway of the coagulation process (Figure 3C). Indeed, the concentration of ULVAN-01 needed to double the coagulation time of the negative control (117 µg/mL) was more than 40-fold higher than that of ULVAN-02 (2.6 µg/mL) (Table 3). However, ULVAN-02 activity on this coagulation pathway was slightly lower than that of Lovenox^®^, which was not the case on the other coagulation pathways. Thus, for the common pathway, the chemical sulfation procedure led to a significantly increased anticoagulant activity of *U. rigida* ulvan.

All these clotting time assays tended to show that the anticoagulant activity of ULVAN-02 was mostly oriented towards the early steps of the coagulation cascade, both on the intrinsic and extrinsic pathways, since its activity on the common pathway, which is the final coagulation with fibrin complex formation (red thrombus), was low compared to both Lovenox^®^ and UFH.

Finally, the anticoagulant activity of the ulvan fractions ULVAN-01 and ULVAN-02 was assessed on two central enzymes of the coagulation process, factors Xa and IIa (also called thrombin). The anticoagulant activity was measured in the presence of antithrombin, a coagulation inhibitor, since UFH and Lovenox^®^ are known for their antithrombin-mediated anticoagulant activity.

**Figure 4:**
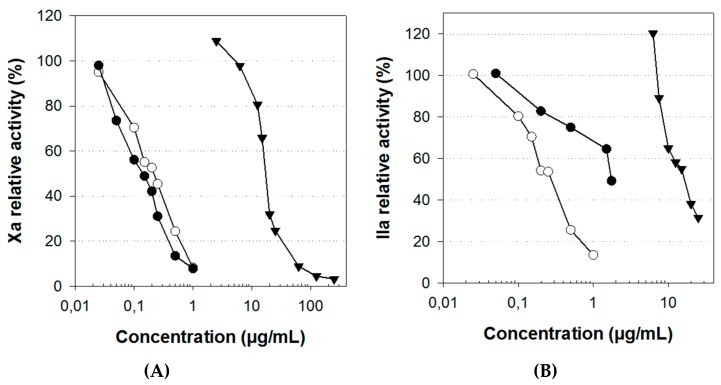
Dose-response curves of antithrombin-mediated (**A**) anti-Xa and (**B**) anti-IIa activities of ULVAN-02 (▼), unfractionated heparin (UFH) (○), and Lovenox^®^ (●). Data shown as the mean, n = 6 (error bars are not indicated for better readability).

ULVAN-01 was devoid of any antithrombin-mediated anti-Xa activity (no activity at 1000 µg/mL, data not shown) while ULVAN-02 showed a maximal activity at a concentration of 100 µg/mL (Figure 4A). However, despite a much higher antithrombin-mediated anti-Xa activity of ULVAN-02 compared to that of ULVAN-01, this activity was 100-fold lower than that of UFH and Lovenox^®^. Indeed, factor Xa residual activity was of 50% with UFH, Lovenox^®^, and ULVAN-02 concentrations of 0.2 µg/mL, 0.125 µg/mL, and 17.5 µg/mL, respectively (Table 3). The lack of antithrombin-mediated anti-Xa activity of the native ulvan fraction ULVAN-01 is in line with the study by Mao et al. [39], which has concluded that rhamnan sulfates from *Monostroma nitidum* have a very low antithrombin-mediated anti-Xa activity (concentration higher than 1000-fold than that of heparin for the same activity). Two sulfated rhamnan extracts were compared by these authors, with MW of 70 kDa and 870 kDa, and sulfate contents of 34.4 and 28.2%, respectively. Although their sulfate content was much higher than that of ULVAN-01 or ULVAN-02 ulvans (11 and 20%, respectively), these sulfated polysaccharides were not more active. Moreover, despite its sulfate content being about 65% lower than that of these sulfated rhamnans described by Mao et al., the concentration of ULVAN-02 needed to achieve a similar antithrombin-mediated activity was 10-fold lower. The work by Majdoub et al. on sulfated rhamnans (20% of sulfates) from the cyanobacteria *Arthrospira platensis* (formerly *Spirulina platensis*) also showed a lack of anti-Xa activity of this type of sulfated polysaccharide [58]. Conversely, Matsubara et al. highlighted the anticoagulant anti-Xa activity of sulfated galactans extracted from the green seaweed *Codium cylindricum*, containing 89% of galactose and only 13.1% of sulfates, and found an activity very similar to that of ULVAN-02 [33]. In conclusion, the degree of sulfation seems to play a key role in the antithrombin-mediated inhibition of factors Xa and IIa by ulvans, but it does not seem to be the only structural feature that drives this reactivity when it comes to polysaccharides from other seaweeds.

In addition, ULVAN-02 was also shown to exhibit an antithrombin-mediated anti-IIa activity (Figure 4B), with a concentration of 18 µg/mL needed to reduce the enzymatic activity of factor IIa by 50%, while ULVAN-01 had no activity (data not shown). Nevertheless, this activity was quite low, 70-fold and 10-fold lower than those of UFH and Lovenox^®^, respectively (Table 3). Lovenox^®^ activity was thus much lower than that of UFH, which is consistent with the known anticoagulant effect of Lovenox^®^ since it mainly targets factor Xa. These results are also in accordance with those obtained with other sulfated rhamnans from green macroalgae, which are able to inhibit thrombin in the presence of antithrombin, but to a lesser extent than heparin. Rhamnose-rich polysaccharides from *Monostroma nitidum* [39] or *Arthrospira platensis* (formerly *Spirulina platensis*) [55]*,* and ulvans from *Ulva* species [43], are, for instance, 100-fold and 10-fold less active than UFH, respectively.

All together, these results confirm previous findings showing that the anticoagulant activity of macroalgal sulfated polysaccharides is affected by their degree of sulfation [46,47]. Indeed, almost all the tests showed a highly significant increase in the anticoagulant activity of *U. rigida* ulvan after chemical sulfation. The results obtained with ULVAN-02 on the PT test are of particular interest as they are not in line with previous studies on sulfated polysaccharides from green macroalgae that have concluded that sulfated polysaccharides have a positive effect on the intrinsic and/or common coagulation pathways, based on the APTT test, while they do not exhibit any activity on the extrinsic coagulation pathway, based on the PT test. For instance, an ulvan from *Capsosiphon fulvescens* (15.4% sulfate content) [59], sulfated rhamnans from *Monostroma latissima* (21–26% sulfate content) [38,40,41] or from *Monostroma nitidum* (28.2–34.4% sulfate content) [39], did not show any activity on the extrinsic pathway, whereas their sulfate content was very close to that of ULVAN-02. Thus, the degree of sulfation does not seem to be the only parameter affecting the anticoagulant activity of ulvans. Other polyanionic polysaccharides from various origins have been shown to have an anticoagulant activity strongly related to their degree of sulfation ([43,44,46,47]: sulfated fucans, in particular, have been extensively studied and their activity has been proved to be mainly mediated by thrombin inhibition by either antithrombin or heparin cofactor II, at different extents. However, numerous studies on sulfated fucans and galactans [60,61,62], and more recently on carrageenans [63], also established that anticoagulant activity, particularly in terms of efficiency, is not merely a function of charge density and depends critically on other structural features [46,47]: monosaccharide composition, glycosidic bounds, MW [50], branching residues, and position of sulfate groups on the sugar backbone [18,60]. Among them, the most important seem to be the sulfation pattern and monosaccharide composition [18,46,47,60]. According to Melo et al. [60], the anticoagulant activity of sulfated galactans is achieved mainly through potentiation of plasma cofactors, including antithrombin, which are the natural inhibitors of coagulation proteases but the structural basis of the interactions involved is very complex, due to the heterogeneity of these polysaccharides. Their study on interactions with thrombin and its cofactors, in particular, notably showed that sulfated galactans can link with antithrombin but require significantly longer chains than heparin to interact with the antithrombin/thrombin complex, and bind to a different site. In addition, Becker et al. highlighted, by molecular modeling techniques, that similarities obtained in the glycosidic linkages and predominant ^1^C_4_ chair form of sulfated fucans and galactans could fully explain their specific interaction with antithrombin and the differences between their anticoagulant activity and that of heparin [62]. All these data indicate that the action mechanism of heparin mimetics including sulfated polysaccharides from marine seaweeds differs from that of heparin, which is well in accordance with the overall results obtained in the present study. It was indeed shown that ULVAN-01 was very slightly active on the intrinsic and common pathways, and inactive on the extrinsic pathway of the coagulation cascade, based on the PT test. It was also inactive on the coagulation factor Xa, which is the upstream common point for intrinsic and extrinsic coagulation pathways. On the contrary, ULVAN-02 was active on all tested pathways, showing that chemical sulfation strongly enhanced the anticoagulant activity of ULVAN-01 and could even enable it to inhibit the antithrombin/Xa or antithrombin/IIa complexes. It was also demonstrated by UHPLC-HRMS analyses that the chemical sulfation method used to obtain ULVAN-02 led to an ulvan with a very heterogenous sulfation pattern, involving both hyper-sulfated and scarcely sulfated zones within the polysaccharidic structure, which is very different from what is usually found in the native form of ulvans, i.e., a sulfation pattern most exclusively involving the position 3 of rhamnose. The most likely hypothesis to explain the anticoagulant activity of ULVAN-02 lies in the probability that a geometrical match between the polysaccharide and its binding site on coagulation factors is increased by sulfation, the sugar backbone providing the geometric constraints to accommodate the binding site, and the increased negative charge due to sulfation pattern modifications in the hyper-sulfated zones providing higher physical interaction properties, further enhancing the binding capacity of the polysaccharide.

### 2.5. Effect of ULVAN-01 and ULVAN-02 on Cell Viability

**Figure 5:**
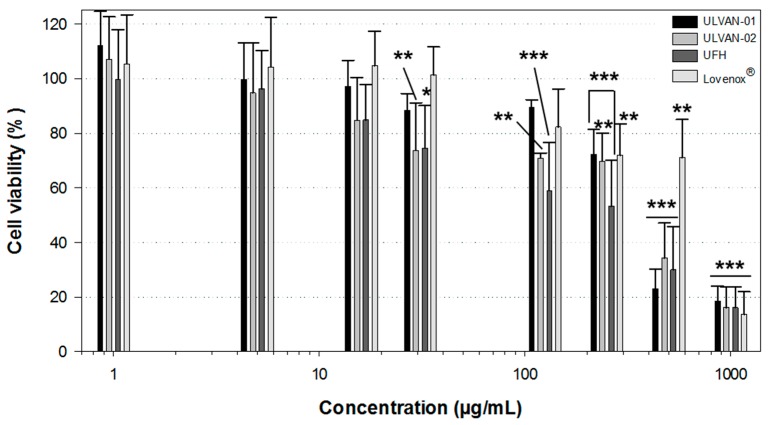
Viability of human fibroblasts treated for 72 h with ULVAN-01, ULVAN-02, unfractionated heparin (UFH), and Lovenox^®^. Results are expressed as the relative percentage of viability compared to the negative control. Data shown as the mean +/−SD, n = 5. Significant differences between samples and negative control are indicated by * (*p* < 0.05), ** (*p* < 0.01), and *** (*p* < 0.001).

The in vitro cytotoxicity of ULVAN-01 and ULVAN-02 was assessed using the MTT assay on human fibroblastic cells, and compared to those of UFH and Lovenox^®^ (Figure 5).

Results showed that ULVAN-01, ULVAN-02, and UFH had a very similar dose-response effect on cell viability. Up to 250 µg/mL, they had a very limited impact on cell viability: about 80% viable cells were indeed enumerated, compared to the negative control. Lovenox^®^ even seemed to have slightly less impact on cell viability. More importantly, ULVAN-02 was absolutely not cytotoxic to human fibroblasts at concentrations within the range of 1–15 µg/mL, corresponding to the concentration range where it was shown to be bioactive, regardless of the anticoagulant activity tested, which is an essential prerequisite to consider any further development of this sulfated ulvan fraction extracted from *U. rigida* as part of a therapeutic anticoagulation application.

## 3. Materials and Methods

### 3.1. Materials

Green macroalga *U. rigida* was cultivated in a pond in the “Ferme du Douhet,” a marine farm in Ile d’Oléron (France). It was collected in 2012 and dried.

UFH was purchased from Interchim (Montluçon, France) (heparin sodium salt 12865E, batch 201274) and Lovenox^®^ was kindly provided by the the Saint-Louis Hospital (La Rochelle, France).

Unless otherwise stated in the text, all chemical reagents were purchased from Merck (Darmstadt, Germany).

### 3.2. Methods

#### 3.2.1. Extraction and Purification Process

First, 500 g of *U. rigida* thallus were washed in 15 L of distilled water (1/30 (*w/w*)) for 10 min at room temperature (RT) and wrung with a fabric cone to remove as much water as possible [48]. The washed algae were then ground at 80 °C in a blender, in 5 L of deionized water, until obtaining 2-mm particles. The 5 L of minced algae in water were transferred into a thermostated tank at 80 °C containing 2.5 L of distilled water. Extraction was processed under constant agitation with a bladed stirrer at a rotation speed of 10 spins/min for 2 h. The pulps were then removed from the tank and filtered with the fabric cone to collect the aqueous extract. The aqueous extract was then recovered and filtered with an ultrafiltration unit equipped with a 15 kDa Kerasep KBW membrane (Novasep Process, Pompey, France). Filtration was carried out at 80 °C at a pressure of 5 bars and a circulation flow of 450 L/h (circulation speed of 5 m/s), until obtaining a retentate around 4°Bx. Next, 1.5 L of retentate was demineralized by passage on a column containing 100 mL of Amberlite FPA 98, a strong anionic resin in the OH^−^ form, in series with a column containing 200 mL of Amberlite IR 120 Na, a strong cationic resin in the H^+^ form. Circulation was processed with a peristaltic pump at a flow rate of 2 BV/h. The deionized product was finally decanted in a water bath at 80 °C for 2 h. After centrifugation at 5000× *g* for 15 min at RT, the fraction, referred to as ULVAN-01, was neutralized to pH 7 with 1 M NaOH and lyophilized.

#### 3.2.2. Sulfation Procedure

The sulfation procedure was carried out on the ULVAN-01 fraction using the sulfur-trioxide pyridine complex (SO_3_–pyridine) method, in dimethylformamide (DMF) and pyridine [64].

Briefly, 500 mg of ULVAN-01 were dissolved in a mixture containing 10 mL of DMF and 1.4 mL of pyridine. The mixture was heated to 60 °C and sulfation was then processed by progressive addition of 3 g of SO_3_–pyridine complex for 2 h at 60 °C under constant stirring. The mixture was left for two additional hours at 60 °C under stirring. After cooling of the mixture at RT, it was centrifuged at 5000× *g* for 10 min at 4 °C. The supernatant was then removed and the pellet was dissolved in 5 mL of 2.5 M NaOH, and 45 mL of absolute ethanol were added (final concentration: 90% (*v/v*)). After 12 h at 4 °C under stirring, the precipitate was isolated by centrifugation (10,000× *g*, 10 min, 4 °C) and dissolved in 60 mL of deionized water. The solution was finally dialyzed for seven days against deionized water (cut-off: 1 kDa) and lyophilized. The resulting sulfated fraction was referred to as ULVAN-02.

#### 3.2.3. Chemical Composition

The neutral sugar content was determined according to the phenol-sulfuric method [65], using glucose as a standard. The uronic acid content was measured as described by Bitter and Muir [66]. The protein content was determined using the Bradford protein assay [67]. The sulfate content was obtained using 3-amino-7-(dimethylamino)phenothiazin-5-ium chloride (Azure A), which binds to the sulfated groups in a polysaccharide chain [68,69]. The quantitation method of polyphenols was adapted from the original one [70], using gallic acid as a standard: briefly, 50 µL of Folin–Ciocalteu reagent and 200 µL of 20% sodium carbonate were successively added to 100 µL of sample and the mixture was incubated in the dark for 45 min at RT, prior to absorbance reading at 730 nm. The ash content of ULVAN-01 was quantified after 15 h at 550 °C. The ash content of ULVAN-02 could not be determined due to a lack of material. All analyses were performed at least in triplicate, except the determination of ULVAN-01 ash content (n = 2), and data are thus shown as the mean.

#### 3.2.4. Analysis of the Fractions by High Performance Size Exclusion Chromatography

The structural and quantitative analyses of ulvan fractions were performed using a HPLC 1100 LC/RID system (Agilent technologies, Santa Clara, CA, USA), with two successive exclusion chromatography columns of 30 cm in size: TSK gel 5000 PW and TSK gel 4000 PW (Tosoh Bioscience, Tokyo, Japan). These analyses were carried out at 30 °C, after injection of 20 µL of 1 mg/mL fraction or sample, at a flow rate of 0.5 mL/min of elution buffer (0.1 M ammonium acetate). Products were detected and quantified by differential refractometry using the HP Chemstation software in off-line mode for processing. The standard curve was made using dextran standards from 1000 to 50,000 Da. The number-averaged molecular weight (M_n_), weight-averaged molecular weight (M_w_) and polydispersity index (I) were calculated as follows [71]:

M_n_ = (Σ N_i_ × M_i_)/Σ N_i_
M_w_ = (Σ N_i_ × M_i_^2^)/(Σ N_i_ × M_i_)
I = M_w_/M_n_

where N_i_ is the number of moles of polymer species and M_i_ the molecular weight of polymer species.

#### 3.2.5. Nuclear Magnetic Resonance (NMR) and Ultra High Performance Liquid Chromatography-High Resolution Mass Spectrometry (UHPLC-HRMS)

Carbon (^13^C) NMR analysis of the ULVAN-01 fraction was performed using a JEOL JNMLA400 spectrometer (400 MHz, JEOL, Peabody, MA, USA) in D_2_O solution at a concentration of 50 mg/mL [48].

The monosaccharide composition and oligosaccharide sequences were determined by UHPLC-HRMS. Analyses were carried out using an UHPLC system, “Acquity UPLC H-class,” coupled to a HRMS system, “XEVO G2-S Q-TOF,” equipped with an electrospray ionization source (Waters, Milford, MA, USA). The UHPLC system was formed by a quaternary pump (Quaternary Solvent Manager, Waters) and an automatic injector (Sample Manager-FTN, Waters) equipped with a 10 µL injection loop. Analyses were performed according to the UHPLC and MS parameters given in Table 4.

The analyses of monosaccharide composition were performed after total acid hydrolysis of ULVAN-01. Briefly, 1 mL of 10 mg/mL ULVAN-01 solution in HCL 3 M was prepared and heated at 100 °C for 5 h (total hydrolysis was monitored over-time using reducing sugar assessment by the dinitrosalicylic acid method [72]). After cooling at room temperature, the sample was centrifuged at 10,000× *g* for 10 min and filtrated through 0.22 μm filter, prior to UHPLC-HRMS analysis.

The analyses of oligosaccharide sequences were carried out on a 9 kDa ulvan fraction filtrated through 0.22 μm filter, obtained after solid supported depolymerization of ULVAN-01. Briefly, 100 mL of ULVAN-01 solution at 25 mg/mL were prepared and depolymerized, using a circuit consisting of the ULVAN-01 solution, a peristaltic pump set at a flow rate of 12 mL/min and a column containing 10 mL of AMBERLITE™ FPC23 H resin in the H+ form. The polysaccharide solution and the column were maintained at 80 °C for 19 h. After cooling at room temperature, the samples were filtrated through 0.22 μm filter, prior to UHPLC-HRMS/MS analysis.

#### 3.2.6. Clotting Time Assays

The anticoagulant activity of the different fractions was determined by measuring the APTT, PT, or TT. All the assays were carried out using a Start4 coagulometer and assay kits from Stago (Asnières-sur-Seine, France), according to the manufacturer’s instructions. Briefly, 90 µL of normal human plasma were mixed with 10 µL of 0.9% NaCl solution containing various sample concentrations for each assay. For the APTT assay, 100 µL of APTT assay reagent were added to the mixture prior to incubation for 3 min at 37 °C and addition of 100 µL of 0.025 M CaCl_2_, and the clotting time (APTT) was recorded. For the TT and PT assays, the mixture was first incubated for 2 min (PT) or 1 min (TT) at 37 °C, before adding 200 µL of PT assay reagent (PT) or 100 µL of TT assay reagent (TT), and recording the clotting time. The negative control used in all assays was 0.9% NaCl solution.

#### 3.2.7. Assays of Antithrombin-Mediated Inhibition of Factors Xa and IIa

Stachrom ATIII and Stachrom Heparin kits (Stago, Asnières-sur-Seine, France) were used to assess the antithrombin-mediated inhibition of factors Xa and IIa (thrombin), respectively, according to the manufacturer’s instructions. Antithrombin was diluted 1:2 in 0.1X kit buffer. Then, 25 µL of sample (aqueous solution of ulvan fraction, UFH or Lovenox^®^ at various concentrations) were incubated with 25 µL of antithrombin (0.626 µg/µL) at 37 °C for 30 s. Thereafter, 25 µL of factor Xa or factor IIa (thrombin) (11.25 nKat/mL) were added. After 30 s of incubation, 25 µL of a 3.25 nM solution of factor Xa chromogenic substrate (CBS 31.39; CH_2_-SO_2_-d-Leu-Gly-Arg-pNA, AcOH) or 25 µL of a 1.4 nM solution of factor IIa chromogenic substrate (CBS 61.50; EtM-SPro-ARG-pNA, AcOH) were added. Factor Xa or IIa activities were immediately measured at 405 nm every 6 s for 5 min. The initial velocity was calculated as the slope of the linear segment of the kinetic curve. All assays were performed in 96-well NUNC microplates (Thermo Fisher Scientific, Waltham, MA, USA), using a Fluostar Omega microplate reader (BMG LABTECH, Ortenberg, Germany).

#### 3.2.8. Evaluation of Cell Viability

Normal human dermal fibroblasts (NHDF) were obtained from ATCC (Manassas, VA, USA): product code CCD-1059Sk; ATCC^®^ CRL-2072™, lot number 62062292, from the skin of a 20-year-old woman according to the supplier’s information. Cells were cultured in Dulbecco’s Modified Eagle Medium (DMEM, PAN Biotech, Aidenbach, Germany) supplemented with 10% (*v/v*) fetal bovine serum (PAN Biotech, Dutscher, France) and 1% (*v/v*) antibiotic solution (10,000 U/mL penicillin, 10 mg/mL streptomycin) (PAN Biotech, Aidenbach, Germany), referred to as complete culture medium thereafter. The cells were cultured at 37 °C with 5% CO_2_, in a temperature-controlled, humidified incubator. The cells were grown in 75 cm^2^ surface ventilated Falcon culture flasks (BD Biosciences, Franklin Lakes, NJ, USA) and subcultured by trypsinization (0.05% (*w/v*) trypsin, PAN Biotech, Aidenbach, Germany). The culture medium was changed every 2 or 3 days. Cells were used between the 3rd and 8th passages for the experiments.

The MTT test was used to evaluate cell viability, according to the method described by Mosmann [73]. This colorimetric assay allows assessing cell proliferation, based on the reduction of a tetrazolium salt (3-(4,5-dimethylthiazol-2-yl)-2,5-diphenyltetrazolium bromide, MTT), by living cells. MTT is a yellow salt. In the presence of mitochondrial succinate dehydrogenase produced by active living cells, the tetrazolium ring they contain is reduced and forms a violet product: formazan. The yellow solution becomes purple and the intensity of the purple coloration is proportional to the number of living cells.

Briefly, 100 µL of complete culture medium containing 5 × 10^4^ cells/mL were seeded in 96-well Falcon microplates (BD Biosciences, Franklin Lakes, NJ, USA) and incubated for 24 h. The medium was then removed and 100 µL of samples prepared in DMEM supplemented with 1% (*v/v*) antibiotic solution, or negative control (DMEM supplemented with 1% (*v/v*) antibiotic solution alone) were added into the wells. After 48 h, 25 µL of MTT (5% (*w/v*) in PBS) were added in each well and the plates were incubated for 4 h at 37 °C. The medium was then removed and 200 µL of dimethyl sulfoxide were added into each well. The plates were then incubated for 10 min and absorbance was read at 550 nm using a Fluostar Omega microplate reader (BMG LABTECH, Ortenberg, Germany).

#### 3.2.9. Statistical Analysis

The one-way Anova was applied to determine significant differences between samples and negative control, using Sigma Plot 12.5 (Systat Software Inc., San Jose, CA, USA).

## 4. Conclusions

A solvent- and acid-free procedure was developed to extract and purify ulvan from *U. rigida*, providing an ulvan fraction of high purity (ULVAN-01), with less than 4% of contaminant proteins remaining at the end of the procedure. The sulfate content of this ulvan was 11%, which is in the lower sulfation range of ulvans extracted from algae of the *Ulva* genus. Data on the anticoagulant activity of ulvans are limited but previous studies focusing on other types of sulfated polysaccharides have shown that sulfation is essential for their anticoagulant activity, although the osidic composition, MW, and sulfate groups position are also important features. To investigate the importance of the degree of sulfation of *U. rigida* ulvan, a chemical sulfation procedure was applied to ULVAN-01, which allowed almost doubling the sulfate content of the native polysaccharide, reaching 20%. The resulting chemically-sulfated ulvan fraction, ULVAN-02, thus exhibited a strongly enhanced anticoagulant activity, regardless of the coagulation pathway tested. Its most significant activity was shown to target the intrinsic and extrinsic pathways, with an activity higher than that of Lovenox^®^ and close to that of UFH. It was indeed less active on the common pathway, although its activity was only slightly lower than that of Lovenox^®^. This last result was confirmed by the low antithrombin-mediated inhibition activity exhibited by ULVAN-02 on two important coagulation factors of the common pathway, Xa and IIa (thrombin), which was also significantly increased compared to ULVAN-01 but remained quite low compared to UFH and Lovenox^®^. Hence, the ULVAN-02 fraction could be a very interesting alternative to heparins, with different targets and a high anticoagulant activity. Moreover, ULVAN-02 did not appear to be cytotoxic on human model cells but further studies are needed to confirm these findings.

## Figures and Tables

**Table 1:** Chemical composition of the ULVAN-01 and ULVAN-02 fractions.

		ULVAN-01	ULVAN-02
Neutral sugars	% (w/w_dry fraction_)	41	33
Uronic acids		34	31
Sulfates on the polysaccharide backbone (sulfate to uronic acid molar ratio)		11 (0.65)	20 (1.30)
Proteins		4	<1
Polyphenols		0	0
Lipids		0	0
Ashes		23	nd ^(d)^
Monosaccharide composition (% molar ratio)	Glc/Gal	12.2	nd ^(d)^
	Xyl	8.0	nd ^(d)^
	Rha	42.6	nd ^(d)^
	GlcN	6.9	nd ^(d)^
	GlcA/IdoA	30.3	nd ^(d)^
M_n_^(a)^ (kDa)		31.3	39.0
M_w_^(b)^ (kDa)		56.7	55.3
I ^(c)^		1.8	1.4

^(a)^ number-averaged molecular weight; ^(b)^ weight-averaged molecular weight; ^(c)^ polydispersity index; ^(d)^ not determined.

**Table 2:** Oligosaccharide sequences identified by ultra high pressure liquid chromatography-mass spectrometry untargeted in-source fragmentation (UHPLC-MS^2^) from ULVAN-01 (sequences 1–4 [48]) and ULVAN-02 (sequences 5–8).

Oligosaccharide Sequence	Ion (m/z)			Ionization	Molecular Formula	Symbolic Representation of Oligosaccharide Fragments ^(a)^ (CFG Nomenclature ^(b)^) 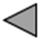 Rha, 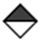 GlcA (or IdoA) ^(c)^ 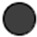 Glc (or Gal) ^(c)^, 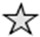 Xyl S = Sulfate Group
	Experimental Value	Predicted Value	Resolution (ppm)			
**1**	1561.3492	1561.3516	1.54	[M − H]^−^	C_54_H_81_O_50_S	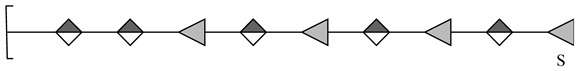
**2**	1561.3492	1561.3516	1.54	[M − H]^−^	C_54_H_81_O_50_S	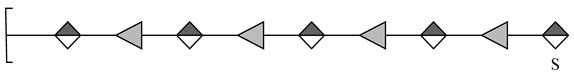
**3**	1723.4083	1723.4045	2.20	[M − H]^−^	C_60_H_91_O_55_S	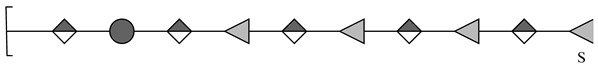
**4**	1839.4554	1839.4518	1.95	[M − H]^−^	C_65_H_99_O_58_S	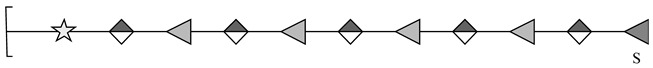
**5**	401.0385	401.0390	1.25	[M − H − H_2_O]^−^	C_12_H_17_O_13_S	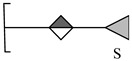
**6**	502.9772	502.9777	0.99	[M − 2H − H_2_O + Na]^−^	C_12_H_16_O_16_S_2_Na	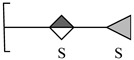
**7**	604.9165	604.9165	0.00	[M − 3H − H_2_O + 2Na]^−^	C_12_H_15_O_19_S_3_Na_2_	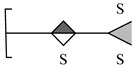
**8**	706.8561	706.8553	1.13	[M − 4H − H_2_O + 3Na]^−^	C_12_H_14_O_22_S_4_Na_3_	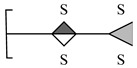

^(a)^ Identified ions correspond to oligosaccharide fragments derived from untargeted fragmentation in the ionization source of the mass spectrometer; ^(b)^ Consortium for Functional Glycomics. Symbolic representations were obtained using Glycoworkbench software [52]; ^(c)^ GlcA and IdoA residues, as well as Glc and Gal, are isomers and therefore cannot be distinguished by MS analysis: symbols used for GlcA et Glc can thus correspond to IdoA and Gal, respectively, two monosaccharides that can be found in ulvans but in smaller proportions.

**Table 3:** Overall analysis of the anticoagulant activity of ULVAN-01 and ULVAN-02.

-	Concentration (µg/mL)			
	Unfractionated Heparin (UFH)	Lovenox^®^	ULVAN-01	ULVAN-02
**Activated partial thromboplastin time (APTT)** ^(a)^	0.75	4.7	500	2.4
**Prothrombin time (PT)** ^(a)^	14.25	480	inactive	45
**Thrombin time (TT)** ^(a)^	0.45	1.82	117	2.62
**Anti-Xa** ^(b)^	0.2	0.125	inactive	17.5
**Anti-IIa** ^(b)^	0.25	1.75	inactive	18

^(a)^ Sample concentration needed to double the clotting time compared to the negative control (0.9% NaCl); ^(b)^ sample concentration for which the enzyme maintained 50% of residual activity.

**Table 4:** Ultra high pressure liquid chromatography (UHPLC) and mass spectrometry (MS) parameters used to determine the monosaccharide composition and oligosaccharide sequences.

	Monosaccharide Composition	Oligosaccharide Sequences
**UHPLC Parameters**		
Column	“Acquity UPLC BEH Amide” (Waters) (2.1 × 500 mm, 1.7 µm), maintained at 30 °C	
Flow rate (µL/min)	0–15 min, 130–200; 15–18 min, 200; 18–30 min, 200–130; 30–37 min, 200	200
Gradient: water (A)/acetonitrile (B) + 0.015% (*v/v*) ammoniac	0–15 min, 90–55% B; 15–17 min, 55% B; 17–18 min, 55–90% B; 18–37 min, 90% B	0–3 min, 100% B; 3–3.1 min 100–70% B; 3.1–10 min, 70–50% B; 10–10.5 min, 50–45% B; 10.5–18 min, 45–100% B
Injection	5 µL (7 °C)	
**MS Parameters**		
Mode	ESI^+^ (centroid)	ESI^−^ (centroid)
Source temperature	120 °C	
Desolvation temperature	250 °C	500 °C
Gas flow rate of the cone	50 L/h	
Desolvation gas flow rate	800 L/h	
Capillary voltage	2.5 kV	
Sampling cone voltage	35 V	130 V
Source compensation	80 V	
Acquisition mass range	50–1200 *m/z*	50–2100 *m/z* (down-regulated according to the fragmented ion with a scan time of 0.15 s)
Lock-mass	Leucine Enkephalin (MW = 555.62 Da, 1 ng/µL)	
